# Pyrazolones Potentiate Colistin Activity against MCR-1-Producing Resistant Bacteria: Computational and Microbiological Study

**DOI:** 10.1021/acsomega.2c07165

**Published:** 2023-02-20

**Authors:** Chonnikan Hanpaibool, Natharin Ngamwongsatit, Puey Ounjai, Sirilata Yotphan, Peter Wolschann, Adrian J. Mulholland, James Spencer, Thanyada Rungrotmongkol

**Affiliations:** † Center of Excellence in Biocatalyst and Sustainable Biotechnology, Department of Biochemistry, Faculty of Science, Chulalongkorn University, Bangkok 10330, Thailand; ‡ Department of Clinical Sciences and Public Health, Faculty of Veterinary Science, Mahidol University, Nakhon Pathom 73170, Thailand; § Laboratory of Bacteria, Veterinary Diagnostic Center, Faculty of Veterinary Science, Mahidol University, Nakhon Pathom 73170, Thailand; ∥ Department of Biology, Faculty of Science, Mahidol University, Bangkok 10400, Thailand; ⊥ Center of Excellence on Environmental Health and Toxicology, Office of Higher Education Commission, Ministry of Education, Bangkok 10400, Thailand; # Center of Excellence for Innovation in Chemistry (PERCH-CIC), Department of Chemistry, Faculty of Science, Mahidol University, Bangkok 10400, Thailand; ∇ Institute of Theoretical Chemistry, University of Vienna, Vienna 1090, Austria; ○ Centre for Computational Chemistry, School of Chemistry, University of Bristol, Bristol BS8 1TS, U.K.; ◆ School of Cellular and Molecular Medicine, University of Bristol, Bristol BS8 1TD, U.K.; ¶ Program in Bioinformatics and Computational Biology, Graduate School, Chulalongkorn University, Bangkok 10400, Thailand

## Abstract

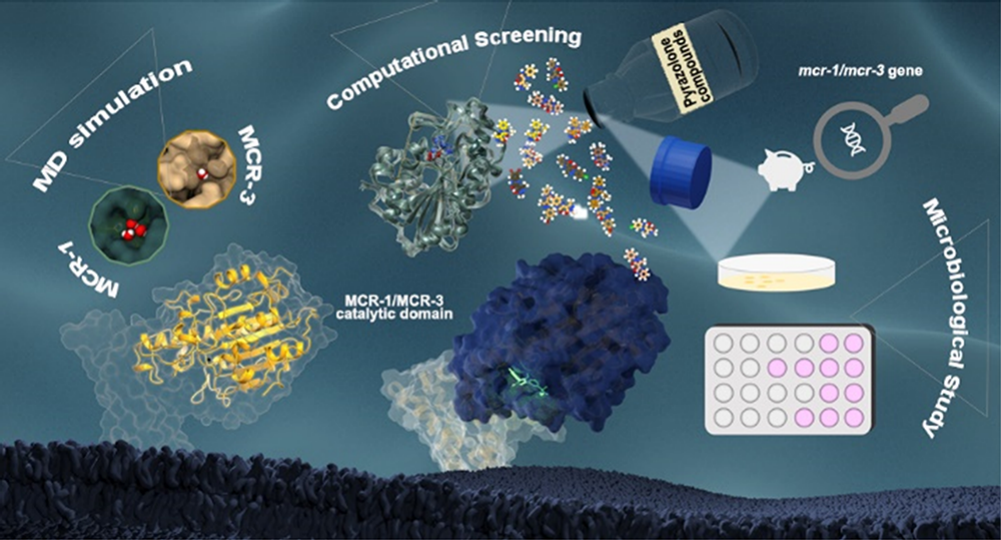

The polymyxin colistin is a last line antibiotic for extensively resistant Gram-negative bacteria. Colistin binding to lipid A disrupts the Gram-negative outer membrane, but mobile colistin resistance (*mcr*) gene family members confer resistance by catalyzing phosphoethanolamine (PEA) transfer onto lipid A, neutralizing its negative charge to reduce colistin interactions. Multiple *mcr* isoforms have been identified in clinical and environmental isolates, with *mcr-1* being the most widespread and *mcr-3* being common in South and East Asia. Preliminary screening revealed that treatment with pyrazolones significantly reduced *mcr-1*, but not *mcr-3*, mediated colistin resistance. Molecular dynamics (MD) simulations of the catalytic domains of MCR-1 and a homology model of MCR-3, in different protonation states of active site residues H395/H380 and H478/H463, indicate that the MCR-1 active site has greater water accessibility than MCR-3, but that this is less influenced by changes in protonation. MD-optimized structures of MCR-1 and MCR-3 were used in virtual screening of 20 pyrazolone derivatives. Docking of these into the MCR-1/MCR-3 active sites identifies common residues likely to be involved in protein–ligand interactions, specifically the catalytic threonine (MCR-1 T285, MCR-3 T277) site of PEA addition, as well as differential interactions with adjacent amino acids. Minimal inhibitory concentration assays showed that the pyrazolone with the lowest predicted binding energy (ST3f) restores colistin susceptibility of *mcr-1*, but not *mcr-3*, expressing *Escherichia coli*. Thus, simulations indicate differences in the active site structure between MCR-1 and MCR-3 that may give rise to differences in pyrazolone binding and so relate to differential effects upon producer *E. coli*. This work identifies pyrazolones as able to restore colistin susceptibility of *mcr-1*-producing bacteria, laying the foundation for further investigations of their activity as phosphoethanolamine transferase inhibitors as well as of their differential activity toward *mcr* isoforms.

## 1 Introduction

The rapid emergence of antimicrobial resistance (AMR) is one of the most important concerns in global public health.^1,2^ It is estimated that, by 2050, the increase in AMR could lead to more than 10 million deaths per year.^3^ A particular problem is antimicrobial resistance in opportunistic bacteria that cause a wide range of infections in both humans and animals.^1,4^ The development of novel drugs active against resistant strains of bacteria is very challenging and normally takes several decades, much slower than the spread of resistant bacterial strains. In recent years, the polymyxin antibiotic colistin has increasingly been exploited as a last-resort treatment in various clinical settings due to the lack of other effective antimicrobials for the treatment of resistant, nosocomial infections.^5−7^ The increase in the use of colistin without proper regulation and the usage of colistin in livestock has resulted in the rise of colistin-resistant bacteria.^8,9^ In addition, colistin use as an agricultural growth promoter is believed to be a major driver of dissemination of mobile resistance.^10^

Colistin exerts its antibiotic function through disruption of the outer membrane of Gram-negative bacteria. The positively charged colistin cyclic peptide head group can bind to negatively charged lipid A and destabilize the integrity of bacterial membrane.^11,12^ Although chromosomally mediated colistin resistance has occasionally been described, recent reports have identified plasmid-mediated colistin resistance in *Enterobacterales*, *Pseudomonas aeruginosa*, and *Acinetobacter baumannii* due to the presence of mobile colistin resistance (*mcr*) genes.^13−18^ MCR catalyzes the transfer of phosphoethanolamine (PEA) onto the phosphate groups of the core disaccharide of lipid A in the outer membrane of Gram-negative bacteria, reducing the overall negative charge on the bacterial membrane and thereby decreasing colistin binding and leading to the development of bacterial resistance.^13,19^

To date, 10 variants of *mcr* genes (*mcr-1* to *mcr-10*) have been discovered^20−23^ and many *mcr* types (*mcr-1* to *mcr*-9) have been isolated from *Escherichia coli* from pigs in Thailand. In one study,^24^ from a panel of 61 *E. coli* isolates, *mcr-1*, *mcr-9*, and *mcr-3* were the most frequently found, in percentages of 40.9, 32.8, and 9.8, respectively. Although the number of *mcr-3*-producing isolates detected was lower than for the other two types, susceptibility testing of *E. coli* harboring the *mcr-3* gene showed that four out of six of isolates had colistin minimal inhibitory concentration (MIC) values above the breakpoint for clinical resistance (>2 μg/mL). Also, our study found that several *mcr* genes, including *mcr-1* and *mcr-3*, as well as the co-occurrence of multiple *mcr* genes (*mcr-1* and *mcr-3*) could be detected in isolates of pathogenic *E. coli* strains from pigs.

**Figure 1:**
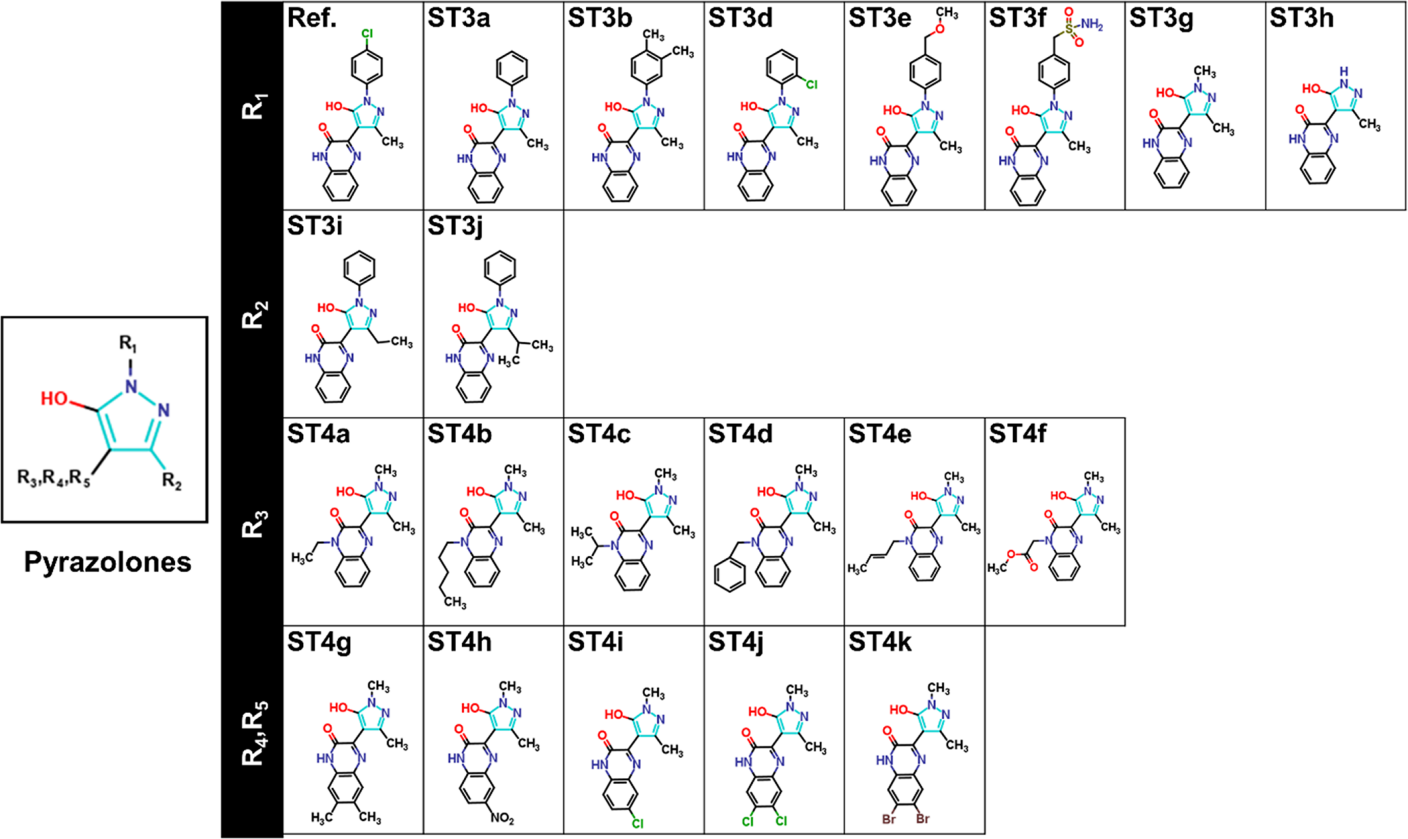
Structures of 21 Pyrazolone Compounds Used in this Study. The reference compound, 3-(1-(4-chlorophenyl)-5-hydroxy-3-methyl-1*H*-pyrazol-4-yl) quinoxalin-2(1*H*)-one (Ref) is shown at the top left.

Pyrazolones found in natural sources have been identified as possessing multiple medically relevant properties including anti-inflammatory and antitumor activities.^25^ Pyrazolone derivatives showed significant antimicrobial activity against several types of Gram-positive and Gram-negative bacteria including *E. coli* strains.^26^ As part of our investigations into the synthesis and biological activities of pyrazolones, we therefore investigated their activity against *mcr*-expressing bacteria. In preliminary experiments, 3-(1-(4-chlorophenyl)-5-hydroxy-3-methyl-1*H*-pyrazol-4-yl) quinoxalin-2(1*H*)-one reduced the colistin MICs of an *mcr-1*-expressing strain in microdilution broth dilution assays. Therefore, we aimed to find novel potentiators of colistin activity from a series of pyrazolones (Figure 1) using structure-based virtual screening against both MCR-1 and MCR-3, each in alternative protonation states generated from molecular dynamics (MD) simulations, verifying activity by *in vitro* assays of antibacterial activity in combination with colistin against *mcr-1*-expressing colistin-resistant bacteria. This work identifies pyrazolone compounds as candidate MCR inhibitors that are able to decrease the colistin minimal inhibitory concentration (MIC) from 8 to 2 μg/mL, i.e., restoring colistin susceptibility in these resistant MCR-1 producing organisms.

## 2 Results and Discussion

### 2.1 Colistin Susceptibility Testing

In order to test candidate pyrazolones for their ability to restore colistin susceptibility to *mcr-*expressing strains, a small of panel of *mcr-1* and *mcr-3* producing *E. coli* was assembled for use in microbroth dilution assays of colistin minimal inhibitory concentration (MIC). Carriage of the *mcr-1* gene in the pathogenic *E. coli* MI907-3bLF strain and of the *mcr-3* gene in the *E. coli* E2LF strain was confirmed by multiplex PCR (Figure S1) with both apparently associated with class 1 integrons (*int1*). To validate susceptibility testing for colistin, *E. coli* with (MI907-3bLF) and without (ATCC 25922 and V13-2LF2) the *mcr-1* gene were tested. According to the EUCAST guidelines,^27^ for *E. coli*, a colistin MIC of ≤2 μg/mL indicates susceptibility or intermediate sensitivity, while resistance is classed as an MIC of >2 μg/mL. The colistin minimal inhibitory concentrations for *E. coli* ATCC 25922 and *E. coli* V13-2LF2 were 0.5 μg/mL, while the MICs of the *E. coli* MI907-3bLF strain carrying the *mcr-1* resistance gene and *E. coli* E2LF (carrying the *mcr-3* gene) were 8 μg/mL. Interestingly, the combination of 8 μg/mL of the reference pyrazolone (3-(1-(4-chlorophenyl)-5-hydroxy-3-methyl-1*H*-pyrazol-4-yl) quinoxalin-2(1*H*)-one) and colistin at 2 μg/mL could inhibit the growth of *E. coli* MI907-3bLF (Table 1). However, at this concentration, the compound had no effect upon the colistin MIC for *E. coli* ATCC 25922 and V13-2LF2 (lacking the *mcr-1* gene). Control experiments (Table S1) also showed no effect on growth of either *mcr*-positive or negative *E. coli* at concentrations up to 64 μg/mL. As the reference pyrazolone showed an ability to potentiate colistin activity against *E. coli* MI907-3bLF (containing the *mcr-1* gene), pyrazolone derivatives were considered for further structure-based virtual screening against the MCR proteins.

**Table 1:** Colistin MIC and Synergistic Effect of 3-(1-(4-Chlorophenyl)-5-hydroxy-3-methyl-1*H*-pyrazol-4-yl)quinoxalin-2(1*H*)-one (Reference Compound) on *Escherichia coli* MI907-3bLF and E2LF Carrying the *mcr-1* and *mcr-3* Genes, in Comparison with *E. coli* ATCC 25922 and V13-2LF2 (Negative Controls)^a^

strains	colistin MIC (μg/mL)	colistin MIC in the presence of reference compound (8 μg/mL)
*E. coli* ATCC 25922 (negative control)	0.5*	1
*E. coli* V13-2LF2 (negative control)	0.5	0.5
*E. coli* MI907-3bLF (*int1^+^*, *mcr-1^+^*)	8	2
*E. coli* E2LF (*int1^+^*, *mcr-3^+^*)	4	4

a *, MIC break point for *E. coli* ATCC 25922 is in the range of *S* ≤ 2 mg/L, *R* > 2 mg/L. *int1^+^* indicates the presence of class 1 integron.^28^

### 2.2 Modeling of the MCR-3 Catalytic Domain

**Figure 2:**
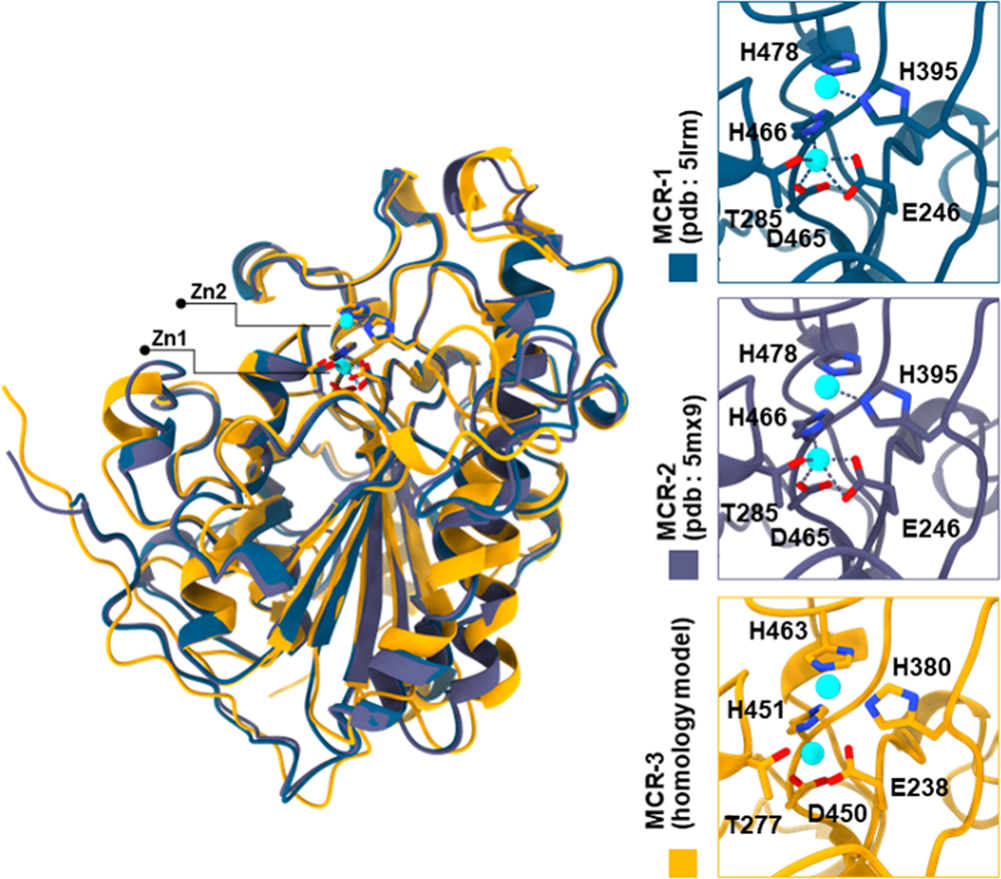
Overall structure of the MCR catalytic domain and active site. Superposition of MCR-3 homology model (gold) and MCR-1/2 crystal structures (blue, magenta). The six conserved residues in the active site (inset) form the zinc-binding motif (Zn1 and Zn2, cyan) in the three paralogues MCR-1^29^ (E246, T285, D465, H395, H466, H478), MCR-2^30^ (E246, T285, D465, H395, H466, H478), and MCR-3 (E238, T277, D450, H380, H451, H463).

Since the *mcr-1* and *mcr-3* genes, as well as co-occurrence of multiple *mcr* genes (*mcr-1* and *mcr-3*) in the same strain, were found in our pathogenic *E. coli* collected from pigs, the MCR-1 and MCR-3 proteins were selected for computational investigation of MCR inhibition by pyrazolones. To date, crystal structures for the catalytic domains of only two MCR isoforms, MCR-1^29^ and MCR-2,^30^ are available in the PDB. In the present study, homology modeling was utilized to predict the 3D structure of the MCR-3 periplasmic catalytic domain to generate a model for use in subsequent computational work. All three homology modeling servers employed (SWISS-MODEL,^31^ Phyre2,^32^ and I-TASSER^33^) selected *Neisseria meningitidis* LptA (a PEA transferase; PDB ID: 4KAY(34)) having 44.8% sequence identity and 63% similarity with MCR-3, as the template on which to build the MCR-3 structure (Figures S2 and S3). The MCR-3 homology model generated by SWISS-MODEL, which contained the highest proportion of residues in the most favored region of the Ramachandran plot, was chosen for use in downstream simulations. By comparison of the MCR-1/2 crystal structures (5LRM^29^/5MX9^30^) and the MCR-3 homology model, six conserved residues (E246 (MCR-1 numbering)/E238 (MCR-3), T285/T277, D465/D450, H466/H451, H395/H480, and H478/H463 (Figure 2)), were identified that coordinate the Zn1 and Zn2 ions within the respective active sites. The importance of Zn^2+^ to MCR activity is demonstrated by the reduction of the colistin MIC of *E. coli* expressing recombinant MCR-1 by up to five dilutions on the removal of Zn^2+^ by treatment with the chelator EDTA.^29^

### 2.3 Structural Dynamics of the MCR-1/MCR-3 Catalytic Domains

**Figure 3:**
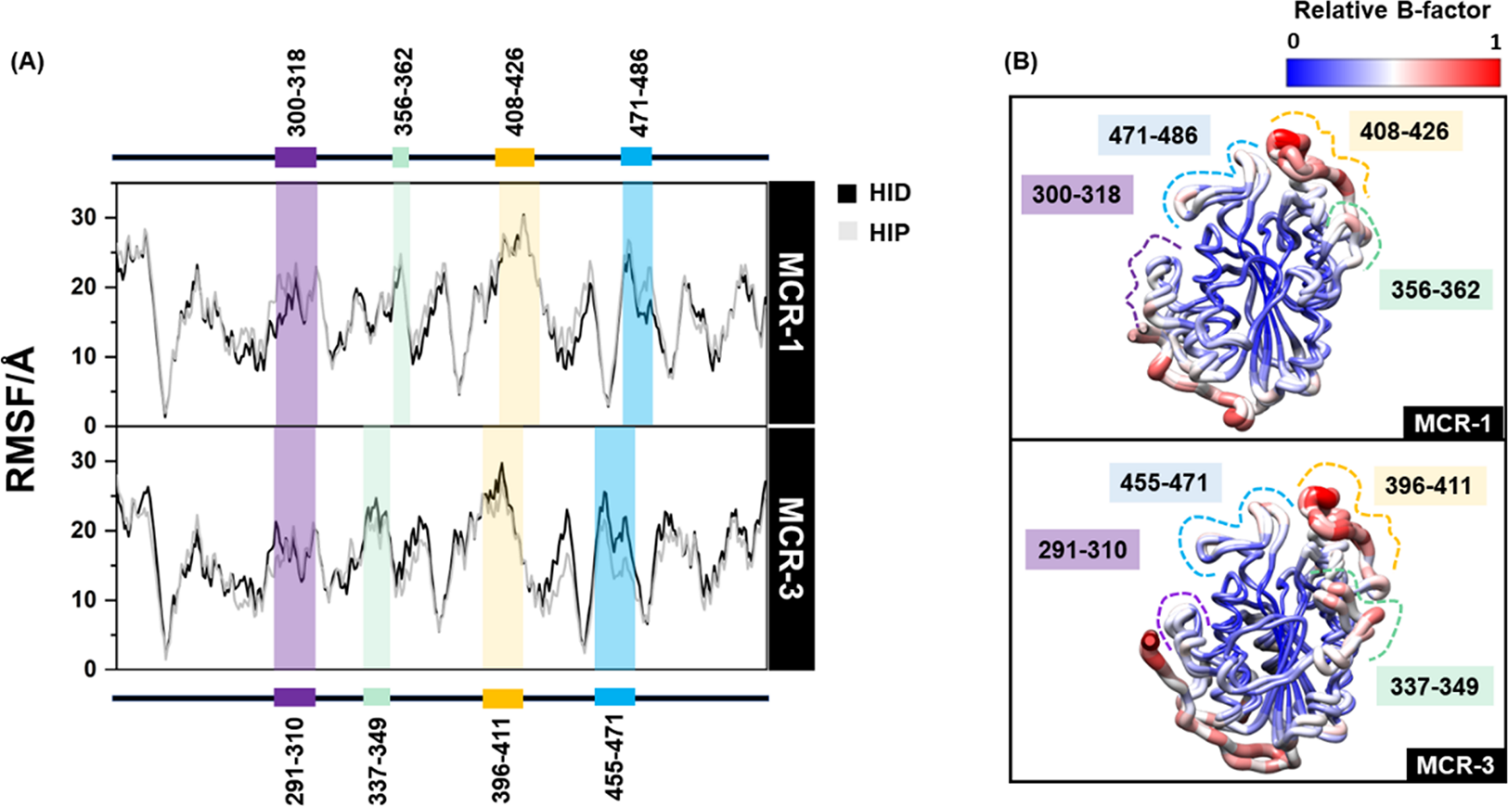
Dynamic behavior of MCR-1/MCR-3 catalytic domains. (A) RMSF values for MCR-1/MCR-3 in the HID and HIP systems averaged across three MD runs (B) Relative B-factors overlaid on representative structures of MCR-1/MCR-3. Flexible loops are highlighted.

To better understand the structure and dynamics of MCR-1/MCR-3, and to identify the most appropriate MCR conformation to use in virtual screening, the catalytic domains of MCR-1 (PDB ID: 5LRM(29)) and MCR-3 (the homology model) were subjected to extended MD simulations. These applied techniques are similar to those applied in our previous studies of MCR-1.^35^ In particular, the behavior and interactions of the two active site histidine residues, H395/H380 and H478/H463, which are highly conserved across MCR isoforms,^23^ (Figure 2) were investigated. Both of these residues are functionally important in MCR-1, with colistin susceptibility increasing when either is mutated.^29^ In addition, in some crystal structures, these two histidine residues coordinate a second zinc equivalent, Zn2, while both could also be involved in stabilizing the bound substrate (phosphoethanolamine; PEA) via interactions with the phosphate oxygen atoms. These different roles in zinc coordination and substrate interaction would require either deprotonation at Nε or protonation at both Nε and Nδ, represented in MD simulations by the HID and HIP systems, respectively. MD simulations were then run on the di-zinc systems with the two histidines in the HID or HIP forms, and the stability of MCR-1/MCR-3 in these systems, encompassing different protonation states of H395/H380 and H478/H463, was considered by analysis of the root mean square deviation (compared to the starting structures for the simulations) and the number of hydrogen bonds. The results showed that all simulations were stable after∼250 ns of the 500 ns simulation, while the number of hydrogen bonds was constant from the beginning of the simulation (Figure S4). Although the fluctuations of MCR-1/MCR-3 in the different protonation states of H395/H380 and H478/H463 were not clearly different, the last 100 ns of each 500 ns simulation was selected for more detailed analysis of their dynamics. As shown in Figure 3, in both proteins, the catalytic site was relatively stable, as seen by low RMSF values, while the four loops (residues 300–318/291–310, 356–362/337–349, 408–426/396–411, and 471–486/455–471) were likely flexible. The overall dynamic behaviors of MCR-1 and MCR-3 are therefore similar to one another.

**Figure 4:**
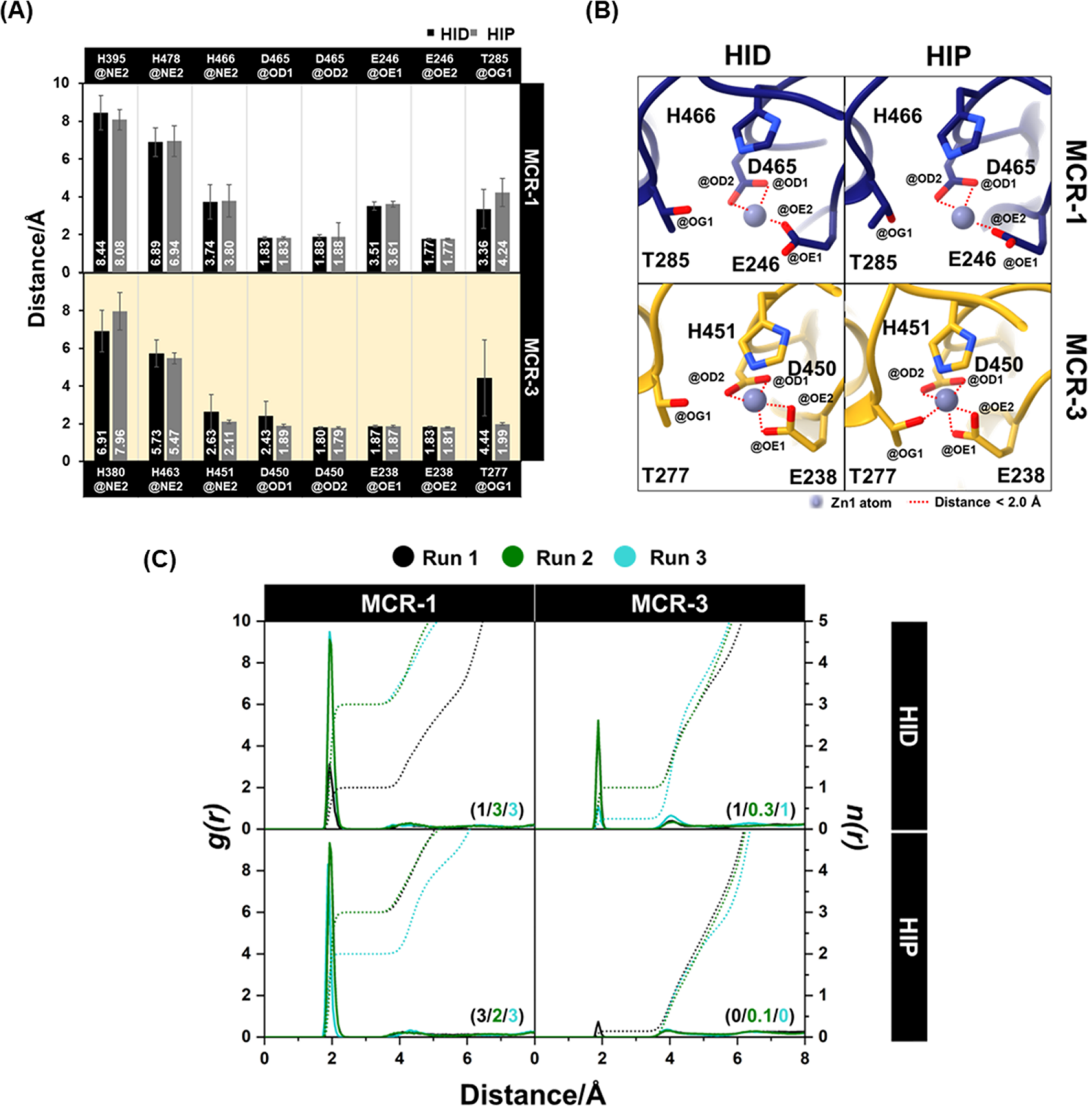
Zn1 Coordination during the last 100 ns of molecular dynamics simulations of MCR-1/MCR-3. (A) Average distances between Zn1 and selected atoms of active site residues. (B) Active site structures from clusters taken from the last 100 ns of MD trajectories based on pairwise best-fit root-mean-square deviations (RMSDs) of MCR-1/MCR-3 in the HID and HIP systems. Zn1-coordinating atoms are shown; dotted lines indicate coordination distances of <2.0 Å. For clarity, water molecules are omitted. (C) Radial distribution function (RDF) of water oxygen atoms surrounding Zn1, where the solid line *g*(*r*) defines the probability of finding a particle at a distance from Zn1 and numbers in brackets represent the average integration number, *n*(*r*) as dotted lines, up to the first minimum determined from the RDF.

To analyze the dynamic behavior of the Zn1 and Zn2 ions in the active sites of MCR-1/MCR-3, the time evolution of the distance between Zn1 and Zn2 in the di-zinc form was calculated. In 500 ns MD simulations of dizinc MCR-1/MCR-3 in both the HID and HIP systems, Zn2 was observed to dissociate from its binding site (H395/H380 and H478/H463) during the heating step at the beginning of the simulation. Dissociation of Zn2 was also found in our previous study of dizinc MCR-1, where Zn2 was seen to dissociate in two out of three 200 ns MD simulations.^35^ In the case of Zn1, the stability of Zn1 was calculated by measuring the distance between the Zn-ligating atoms of coordinating residues (T285/T277, D465/D450, H466/H451, E246/E238) and Zn1 (Figure 4, S5). The results show that for MCR-1/MCR-3 in both the HID and HIP systems, Zn1 was stably bound, maintaining an overall distance to the coordinating ligands (carboxylate oxygens of E246/E238) of ∼1.8 Å along the simulation time.

Altogether, the simulations indicated that in both MCR-1 and MCR-3, the catalytic Zn1 ion remained stably coordinated by six ligands, but that changes in the protonation states of residues H395/H380 and H478/H463 may affect its coordination environment. Analysis of the distances between Zn1 and the surrounding residues found that for the MCR-1 system, there were three and four protein atoms coordinated (with distances less than 2.6 Å) to Zn1 in the unprotonated (HID) and protonated (HIP) systems, respectively. In the MCR-3 system, there were five and six protein atoms strongly coordinated to Zn1 in the unprotonated (HID) and protonated (HIP) systems, respectively (Figure S6). However, the radial distribution function (RDF) of water molecules surrounding Zn1 showed that for MCR-1, one to three water molecules were found within 2 Å of the Zn1 ion in both the unprotonated (HID) and protonated (HIP) systems. No water molecules are present in the active site of MCR-3 in the HIP systems, while either one or two water molecules were located within 2 Å of Zn1 in the HID systems (Figure 4C). In all systems, D465/D450 (of MCR-1 and MCR-3, respectively) showed bidentate coordination with Zn1, while with MCR-1 in the HID and HIP systems, E246 showed monodentate coordination to Zn1. In contrast, MCR-3 E238 in both the HID and HIP systems showed bidentate coordination to Zn1 (Figure 3B). These results must be considered in the light of the known tendency of the LJ12-6 parameter set to favor octahedral Zn^2+^ coordination,^35−38^ which may be achieved by addition of water molecules and/or use of bidentate, rather than monodentate, interactions with carboxylate ligands. Nevertheless, based on this analysis, the RDF then implies that the protonation states of H380 and H463 may affect water accessibility in the MCR-3 active site to a greater extent than is the case for MCR-1.

A previous experimental study from Xu and coworkers,^39^ based on mutagenesis of the Zn binding motif of MCR-3, implicated both H380 and H463 in recognition of the substrate, phosphoethanolamine. Alanine substitutions at H380 and H463 abolished enzyme activity, as measured by colistin MIC and mass spectrometry of lipid A, in *E. coli* expressing MCR-3. This contrasts with results obtained for MCR-1,^29^ where mutation to alanine of H395 completely abolishes activity, whereas that of H478 is reduced but not completely abolished. During the MCR-catalyzed reaction, H380 and H478 in the protonated (HIP) state could be stabilizing the phosphate oxygen of bound PEA. However, in the various crystal structures of MCR catalytic domains, binding of the second (Zn2) metal ion would require both of these residues to be in the unprotonated state. In this study, we found that the stability of the residues H395/H380 and H478/H463 had different patterns (Figure S7). In particular, H395/H380 (ND1, CG, CB, and CA atoms) displayed higher levels of fluctuation than H478/H463 during simulations in both the HID and HIP states in the MCR-1/MCR-3 systems. In contrast to H395/H380, all the simulations clearly suggested that H478/H463 showed little rotational movement over the simulation time. However, the distribution of rotation angles for protonated H380 (MCR-3) showed a wider range (170, 50, −70 to −150 and −170 degrees), when compared to the unprotonated form (where conformers in the 80 and −60 degree orientations only were observed). In contrast, the distributions for H463 were not clearly different between protonated and unprotonated systems. Similar observations were made in MCR-1, in which the H380 equivalent residue H395 exhibited more movement than H478.^35^

### 2.4 *In Silico* Screening of Pyrazolones

**Figure 5:**
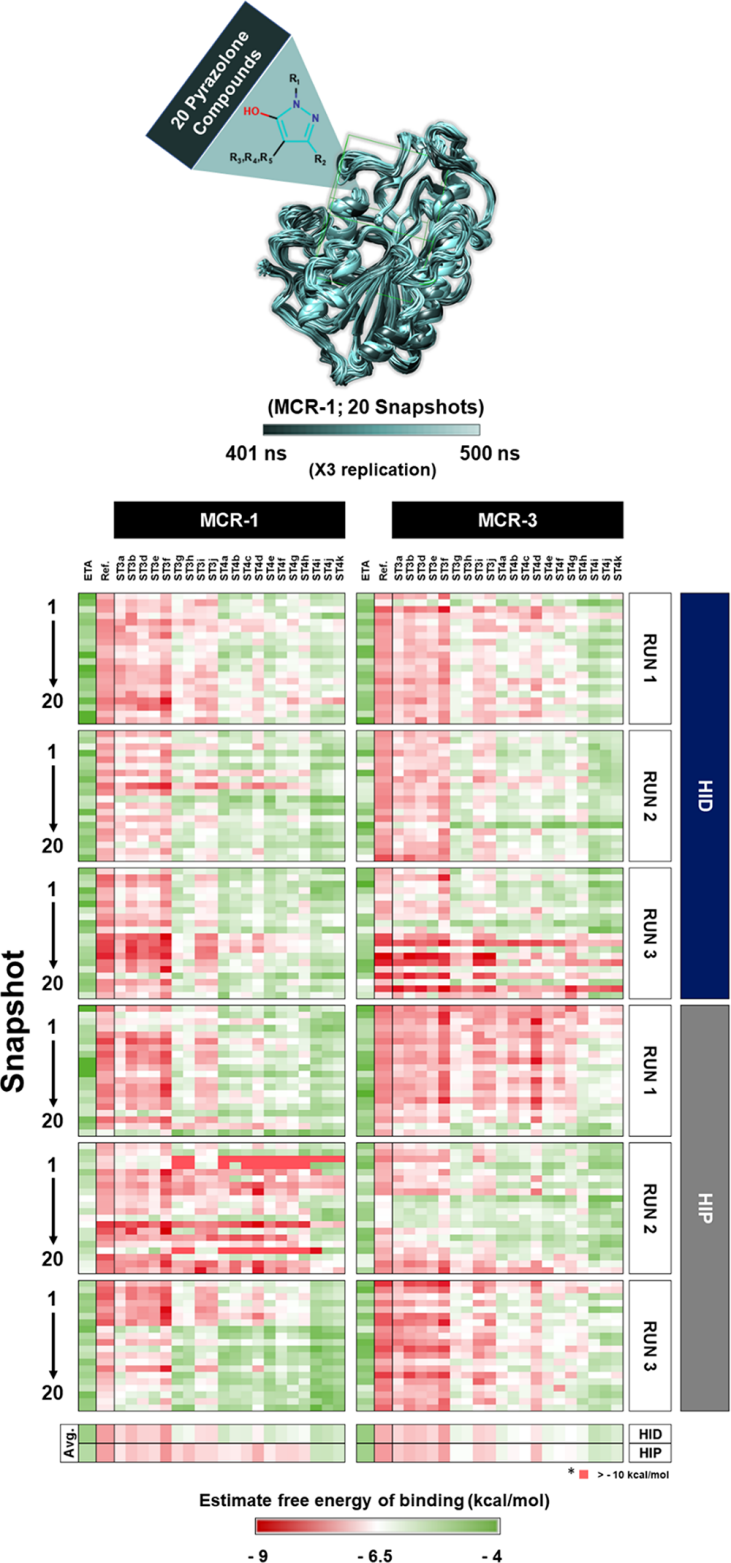
Docking of pyrazolone compounds into MCR-1 and MCR-3. An in-house library of 20 compounds, together with ETA (*X*-axis), was docked into the active sites of MCR-1 and MCR-3 (extracted from the last 100 ns of three independent MD simulations; 20 snapshots per simulation, *Y*-axis) with H395/380 and H478/H463 each in both the HID and HIP states. The free energy of binding is represented in the color bar; with red as the lowest and green as the highest energy.

As demonstrated above, the reference pyrazolone compound reduced the colistin MIC of *E. coli* carrying the *mcr-1* gene. To further explore this finding, an in-house library of compounds containing pyrazolone groups (20 compounds) was selected as a set of candidates for screening for MCR-1 and MCR-3 inhibition. Representative MCR-1/MCR-3 structures (20 snapshots) were extracted from the last 100 ns of three independent MD simulations, and all compounds were docked into the active sites of all twenty MCR-1/MCR-3 structures using Autodock4 (Figure 5). A previous study reported that ethanolamine (ETA) as a substrate analog could inhibit MCR-1 function and that the mode of binding to the active site could be established in the crystal structure of an MCR-1 complex.^40^ Thus, ETA was docked into the MCR-1/MCR-3 active site for use as a further reference compound. The results, obtained from three independent docking runs for each of the 20 MD-derived MCR-1 and MCR-3 structures, yielded a lowest average free energy of binding of −7.1/–7.2 and −7.1/–7.1 kcal/mol and a highest energy of −5.2/–5.3 and −5.2/–5.4 kcal/mol for the set of pyrazolones docked into the HID/HIP systems in MCR-1 and MCR-3, respectively (average values calculated from the total of 60 docking runs for each system (Table S2)). Encouragingly, these data showed that all of the pyrazolone compounds have a free energy of binding to the MCR-1 catalytic domain lower than that obtained for ETA (the lowest average free energies of binding of ETA to the HID/HIP systems in MCR-1 and MCR-3 were −4.4/–4.6 and −4.6/–4.5 kcal/mol, respectively).

### 2.5 Testing Compound Activity

To test compound activity in bacteria, some compounds were selected for testing in combination with colistin for inhibition of colistin resistance in *E. coli*. From the docking results with the reference pyrazolone compound, for which the average binding free energies were −6.5/–6.6 and −6.5/–6.6 kcal/mol for docking to structures derived from simulations of MCR-1/MCR-3 in the HID and HIP states, respectively, compounds ST3e and ST3f were selected based on their calculated free energies. Docking of ST3e gave similar binding free energies to the reference compound (−6.5/–6.4 and −6.4/–6.5 kcal/mol for the MCR-1/MCR-3 HID and HIP systems, respectively), whereas ST3f gave lower energies (−7.1/–7.1 and −7.2/–7.1 kcal/mol, for MCR-1/MCR-3 in the HID and HIP states, respectively). From the docking results, a possible binding pose of each compound was selected from 1 of the 10 runs with the lowest binding free energy. Residues common to interactions of ST3f, ST3e, and the reference compound with MCR-1 were E246, T285, H395, H466, H478, T283, S284, G479, M480, and P481, which collectively participated in both hydrophobic and hydrogen bonding interactions. Interestingly, the first five of these residues identified as involved in interaction with pyrazolones were already identified as important to MCR-1 activity.^19^

The results from MIC testing of the combination of colistin with the reference pyrazolone and compounds ST3e and ST3f are shown in Table 2. In the absence of pyrazolones, the colistin minimum inhibitory concentration for *E. coli* ATCC25922 and *E. coli* V13-2LF2 was 0.5 μg/mL, while for strains carrying the *mcr-1* gene, *E. coli* MI907-3bLF, *E. coli* MI951-bLF/62, and *E. coli* E5-2LF, the colistin MIC was 8 μg/mL. For the strain carrying only the *mcr-3* gene, the colistin MIC was 4 μg/mL. In the presence of the pyrazolones (ST3f and ST3e) at 8 μg/mL, the colistin minimum inhibitory concentrations for *E. coli* ATCC25922 and *E. coli* V13-2LF2 were 0.5–2 μg/mL, while for the strain carrying the *mcr-1* gene, *E. coli* MI907-3bLF, the colistin MICs were 2 and 4 μg/mL, respectively. For the co-occurrence of multiple *mcr* genes (*mcr-1* and *mcr-3*) in *E. coli* MI951-bLF/62 and *E. coli* E5-2LF, the colistin MIC values were 4 μg/mL. The same result was obtained when the pyrazolone compounds were tested against a strain carrying the *mcr-3* gene only (*E. coli* E2LF). In the absence of colistin compounds, ST3e and ST3f showed no effect on growth of either *mcr*-positive or *mcr*-negative *E. coli* at concentrations up to 64 μg/mL (Table S1); none of the additional pyrazolones tested for potentiation of colistin activity (compounds ST3b, ST4j, ST3j) showed effects of greater than one dilution on colistin MIC (Table S3).

**Table 2:** Effect of Pyrazolone Compounds (8 μg/mL) on Colistin MICs of *Escherichia coli* ATCC 25922, V13-2LF2 (Negative Control), MI907-3bLF (*mcr-1*), MI951-bLF/62 (*mcr-3*) E2LF, and E5-2LF (*mcr-1*, *mcr-3*) Carrying the *mcr-1* and *mcr-3* Genes^a^

		colistin MIC in the presence of compound (8 μg/mL)		
strain	colistin MIC (μg/mL)	reference compound	ST3f	ST3e
*E. coli* ATCC 25922 (negative control)	0.5*	1	0.5	1
*E. coli* V13-2LF2 (negative control)	0.5	0.5	1	2
*E. coli* MI907-3bLF (*int1^+^*, *mcr-1^+^*)	8	2	2	4
*E. coli* MI951-bLF/62 (*mcr-1^+^*, *mcr-3^+^*)	8	4	4	4
*E. coli* E2LF (*int1^+^*, *mcr-3^+^*)	4	4	4	4
*E. coli* E5-2LF (*int1^+^*, *mcr-1^+^*, *mcr-3^+^*)	8	4	4	4

a *, MIC break point for *E. coli* ATCC 25922 is in the range of *S* ≤ 2 mg/L and *R* > 2 mg/L.

### 2.6 Binding Interactions of Pyrazolones with MCR-1

**Figure 6:**
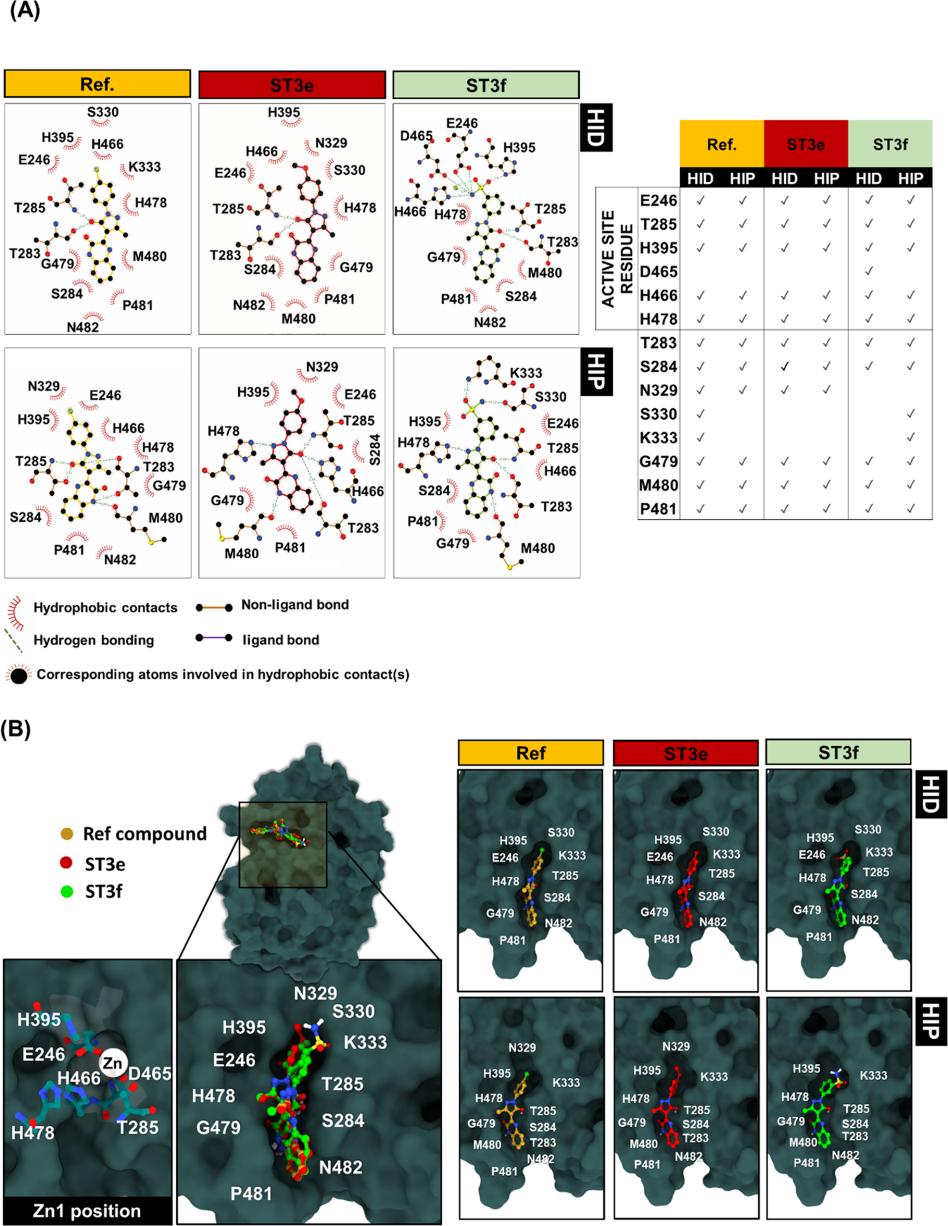
Interactions of pyrazolones with MCR-1. (A) Ligplot analyses of interactions of docked compounds (Ref, ST3e, and ST3f, lowest energy poses) with the MCR-1 active site showing hydrophobic and hydrogen bond contacts with surrounding residues (listed right, ticks indicate interactions); (B) binding poses overlaid on the space-filling model of the MCR-1 active site.

Experimental testing of the two selected pyrazolones ST3e and ST3f identified that 8 μg/mL of either compound can reduce the colistin concentration required to inhibit the growth of the *E. coli* strain MI907-3bLF containing the *mcr-1* gene, in the case of ST3f by two dilutions, while the growth of the two control strains lacking the *mcr-1* gene was not affected. These results suggest that pyrazolones may bind to the MCR-1 active site, resulting in inhibition of the MCR-1 function. The MCR catalytic zinc ion is coordinated by four residues: E246/E238 (carboxylate oxygens), T285/T277 (hydroxyl oxygen), D465/D450 (carboxylate oxygens), and H466/H451 (imidazole nitrogen, in the HIP system). However, during MD simulations of both the MCR-1 (HID and HIP) and MCR-3 (HID) systems, Zn^++^ coordination by the key residue T285/T277 (the site of phosphoethanolamine addition during the catalytic cycle) was replaced by a water molecule. To investigate the interactions of pyrazolones with MCR-1, the orientations of the two selected compounds ST3e and ST3f and of the reference compound, bound with the lowest free energy as estimated from docking results, were input to the Ligplot server.^41^ The orientation of all three docked pyrazolone compounds revealed that they shared common interactions with essential residues within the MCR-1 active site, E246, T285, H395, H466, and H478, in both the HID and HIP systems (Figure 6). In addition, residue T285 made a H-bond interaction with the oxygen atom of the pyrazolone ring. The remaining active site residues made hydrophobic interactions with the bound pyrazolone. In contrast, when the compounds were docked into the MCR-3 active site, only residue T277 made common interactions with the reference compound and compounds ST3e and ST3f (Figure S8) and in the lowest energy poses, the pyrazolone scaffold was bound in a different orientation to that observed in MCR-1 (Figure S9).

In addition, the crystal structure of the MCR-1 catalytic domain in complex with a substrate analog (d-glucose) determined in a previous crystallographic study^40^ was investigated to compare pyrazolone binding with the likely binding mode of the lipid A substrate. This analysis revealed that residues T283, S284, Y287, P481, and N482 define a binding pocket for MCR substrates in the active site near T285. Specifically, d-glucose was held in place by residues T283, S284, and N482. Docking of the three pyrazolones (reference compound, ST3e, and ST3f) into the MCR-1 active site revealed all three to make interactions with these residues (T283, S284, and N482). Consistent with the lack of activity of pyrazolones against *E. coli* strains carrying the *mcr-3* gene, these interacting residues are not found in MCR-3. Thus, while future experimental validation will be necessary to confirm the orientations of pyrazolones bound to the MCR-1 active site, the results of docking experiments are consistent with available structural information on the likely mode of lipid A binding to MCR-1 and with the differing activities of pyrazolones toward MCR-1 and MCR-3 expressing *E. coli*.

## 3 Conclusions

In the case of MCR-1 inhibitor screening, results from colistin MIC assays demonstrate that selected pyrazolone compounds can decrease the colistin concentration required for growth inhibition of MCR-1 expressing *E. coli* from 8 to 2 μg/mL. Hence, pyrazolones could represent candidate MCR-1 inhibitors that exploit interactions with T285, a residue important to substrate binding as the site of phosphoethanolamine addition during the MCR-1 catalytic cycle. In addition, the docked pyrazolones make similar interactions within the MCR-1 active site, involving residues T283, S284, and N482, to those made by the substrate analog d-glucose as described in a previous study.^40^ These data suggest that the pyrazolone scaffold is able to interact with the MCR active site in a manner that replicates aspects of binding of the lipid A substrate, supporting further exploration of pyrazolones as components of colistin combinations able to overcome MCR-1-mediated resistance. Moreover, our simulations indicate subtle but potentially important differences between MCR-1 and MCR-3 in the active site structure and pyrazolone interaction that may help to explain the experimentally observed differences in behavior. The differential effects of pyrazolones on colistin susceptibility of *E. coli* expressing MCR-1 and MCR-3 indicate that interactions of MCR proteins with candidate inhibitors may be isoform-specific and justify inclusion of multiple isoforms in future investigations of MCR inhibition.

## 4 Methods

### 4.1 Antimicrobial Susceptibility Testing

*E. coli* strains collected from pig farms were obtained from the Faculty of Veterinary Science, Mahidol University. Six *E. coli* strains (ATCC 25922, V13-2LF2, MI907-3bLF, MI951-bLF/62, E2LF, and E5-2LF) were used in this study. *E. coli* ATCC 25922 and V13-2LF2 were used as negative controls. The *mcr-1*–*3* genes were detected by multiplex PCR^42^ (Figure S2). *E. coli* MI907-3bLF and E2LF are positive for *mcr-1* and *mcr-3*, respectively, while *E. coli* MI951-bLF/62 and E5-2LF show coexistence of *mcr-1* and *mcr-3.* Colistin was purchased from Sigma-Aldrich. Pyrazolone compounds were synthesized as described previously.^43^ Susceptibility testing was performed using the broth microdilution procedure according to the European Committee on Antimicrobial Susceptibility Testing (EUCAST) recommendation. *E. coli* colonies were selected from Brain Heart Infusion (BHI) agar and transferred to BHI broth to grow bacteria to OD_600_ = 0.08–0.1 (1 × 10^8^ CFU/mL) and were then transferred to Mueller-Hinton broth for minimum inhibitory concentration (MIC) testing at a final cell concentration of 5 × 10^5^ CFU/mL. In the case of colistin, its final concentration was in the range of 0.0625 to 64 μg/mL in 2-fold dilutions. For synergism testing, pyrazolone compounds were added at a final concentration of 8 μg/mL with final colistin concentrations tested in the range of 0.5 to 16 μg/mL in 2-fold dilutions. All bacterial strains were incubated at 35 ± 1 °C for 18 ± 2 h. The MIC values were detected by the resazurin assay.^44^

### 4.2 Preparation of MCR-3 Structure

The MCR-3 structure was modeled based on sequence accession no. WP_039026394 using the structure of *N. meningitidis* lipooligosaccharide phosphoethanolamine transferase A (LptA, 4KAY.pdb^34^) as a template. MCR-3 models obtained from different software, SWISS-MODEL,^31,45^ Phyre2,^32^ and i-TASSER,^33,38,46^ were then validated by their Ramachandran plots using RAMPAGE^47^ (Figure S3). The selected MCR-3 model, with H380 and H463 in both unprotonated (HID) and protonated (HIP) forms, was set up according to our previous study on MCR-1 and MCR-2. In this study, the MCR-1 structure was obtained from the PDB databank (PDB ID: 5LRM(29)). The protonation states of the remaining ionizable residues were assigned using PROPKA3.1 at pH 7.4.^48,49^ The AMBER ff1S4B force field^50^ was applied for the protein, while the zinc ions were treated using the standard 12-6 Lennard–Jones (LJ) nonbonded model. While such simple nonbonded models have limitations in their description of zinc coordination,^38^ we have tested these in previous MD simulations of MCR-1.^35^ In consequence, we consider this description to provide a useful first approximation for the comparison of MCR-1 and MCR-3 presented here, and use it in the MD simulations presented. The five disulfide bonds and the missing hydrogen atoms in the MCR-3 structure were generated by the LeaP module implemented in AMBER16. Then, the systems containing H380 and H463 in both the HID and HIP forms were solvated by the TIP3P water model with at least 12 Å distance from the solute and neutralized by six and four sodium ions, respectively. Altogether, each system was composed of ∼12,700 TIP3P water molecules in an octahedral box with a volume of ∼475,000 Å^3^.

### 4.3 Molecular Dynamics Simulations and Analysis

The two MCR-1/MCR-3 systems (containing H395/H380 and H478/H463 in both the HID/HID and HIP/HIP forms) were investigated using all-atom MD simulations with three different velocities under periodic boundary conditions using the PMEMD.cuda module in AMBER16^51^ for 500 ns. A cutoff of 10 Å was set for nonbonded interactions, and the particle mesh Ewald (PME) method^52^ was employed for long-range electrostatic interactions. The hydrogen atoms, water molecules, and whole systems were minimized accordingly with 1000 steps of the steepest descent (SD) followed by 8500 steps of the conjugated gradient (CG).^53^ Using a time step of 2 fs, the systems were heated up from 10.0 to 310.0 K for 200 ps and were then equilibrated for another 3500 ps. Afterward, the simulations were carried out with the constant-temperature, constant-pressure (NPT) ensemble at 310.0 K and pressure of 1 atm.^54^ Finally, the systems were simulated for 500 ns, and we obtained a total of 50,000 MD snapshots with equal time spacing over the course of the MD simulation. The MD trajectories from the last 100 ns of the three independent simulations were extracted for analysis. The root-mean-square displacement (RMSD), number of H-bonds, the distance between the Zn^2+^ ion(s) and surrounding residues, the radial distribution function (RDF) of water molecules around the catalytic Zn1, and torsion angle calculations on ND1_CG_CB_CA for H395/H380 and H478/H463 as well as CE1_ND1_CG_CB for T277 along the simulation time were analyzed using the CPPTRAJ module.^55^ Twenty MD snapshots extracted from the last 100 ns of all three independent simulations, with water molecules and neutralizing ions removed, were taken for molecular docking studies.

### 4.4 Virtual Screening

The 3D structures of all 20 pyrazolones, including the reference compound, were built using Gaussview 5.0^56^ in accordance with their pKa values as predicted by Chemaxon (https://www.chemaxon.com). Each pyrazolone was optimized at the HF/6-31G(d) level of theory using Guassian 09.^57^ All 120 MD snapshots of the MCR-1/MCR-3 proteins and the optimized compounds were prepared using a script from ADTools.^58^ For the docking procedure, a grid of (*X*, *Y*, *Z*) = 60, 60, 60 (grid spacing 0.375 Å) centered at the active site Zn1 atom, was generated with AutoGrid and used for calculating atomic affinity maps. All pyrazolones were separately docked into the MCR-1 active site of each snapshot using Autodock4.^58^ The pyrazolone compounds with high binding affinities for MCR-1/MCR-3, as predicted by molecular docking, were then selected for biological testing.

## Supporting Information Available

Figures showing the presence of *mcr-1*/*mcr-3* in *E. coli* strains tested; homology models of MCR-3; sequence alignment of selected PEA transferases; time dependence of RMSD, H-bonding, Zn1 coordination, and flexibility of selected active site residues during MD simulations of MCR-1 and MCR3; interactions of pyrazolones docked into MCR-3; and comparison of pyrazolones docked into MCR-1/MCR-3; tables showing MICs of pyrazolones against *E. coli* strains; average binding free energies for pyrazolones docked into MCR-1/MCR-3; and colistin MICs for *E. coli* strains in the presence of selected pyrazolones; and supplementary references (PDF)

## Notes

Structures of the *mcr*-3 homology model and docked complexes of pyrazolones and input files for all simulations are available at the University of Bristol data.bris Research Data Repository (https://data.bris.ac.uk/data/).
