# Supplementary material for: Pyrazolones Potentiate Colistin Activity against MCR-1-Producing Resistant Bacteria: Computational and Microbiological Study
Source: ACS Omega. 2023 Feb 20;8(9):8366–76. doi: 10.1021/acsomega.2c07165 (PMC9996792; doi:10.1021/acsomega.2c07165)
Supplement: Supplementary file 1 — ao2c07165_si_001.pdf [file ao2c07165_si_001.pdf]

Supporting Information

Pyrazolones Potentiate Colistin Activity against MCR-1-Producing Resistant Bacteria: Computational and Microbiological Study

Chonnikan Hanpaibool[1], Natharin Ngamwongsatit[2,3], Puey Ounjai[4, 5], Sirilata Yotphan[6], Peter Wolschann[7], Adrian J. Mulholland[8], James Spencer[9,*], Thanyada Rungrotmongkol[1,10,*]

[1] Center of Excellence in Biocatalyst and Sustainable Biotechnology, Department of Biochemistry, Faculty of Science, Chulalongkorn University, Bangkok 10330, Thailand

[2] Department of Clinical Sciences and Public Health, Faculty of Veterinary Science, Mahidol University, Nakhon Pathom 73170, Thailand

[3] Laboratory of Bacteria, Veterinary Diagnostic Center, Faculty of Veterinary Science, Mahidol University, Nakhon Pathom 73170, Thailand

[4] Department of Biology, Faculty of Science, Mahidol University, Bangkok 10400, Thailand

[5] Center of Excellence on Environmental Health and Toxicology, Office of Higher Education Commission, Ministry of Education, Bangkok 10400, Thailand

[6] Center of Excellence for Innovation in Chemistry (PERCH-CIC), Department of Chemistry, Faculty of Science, Mahidol University, Bangkok 10400, Thailand

[7] Institute of Theoretical Chemistry, University of Vienna, 1090 Vienna, Austria

[8] Centre for Computational Chemistry, School of Chemistry, University of Bristol, Bristol BS8 1TS, UK

[9] School of Cellular and Molecular Medicine, University of Bristol, Bristol BS8 1TD, UK

[10] Program in Bioinformatics and Computational Biology, Graduate School, Chulalongkorn University, Bangkok 10400, Thailand

*Corresponding authors. Tel: +66 218 5426; Fax: + 66 22185418 (T.R.); Tel: +44 (0) 117 331 2084; Fax +44 (0) 117 331 2091 (J.S.).

E-mail address: thanyada.r@chula.ac.th; jim.spencer@bristol.ac.uk

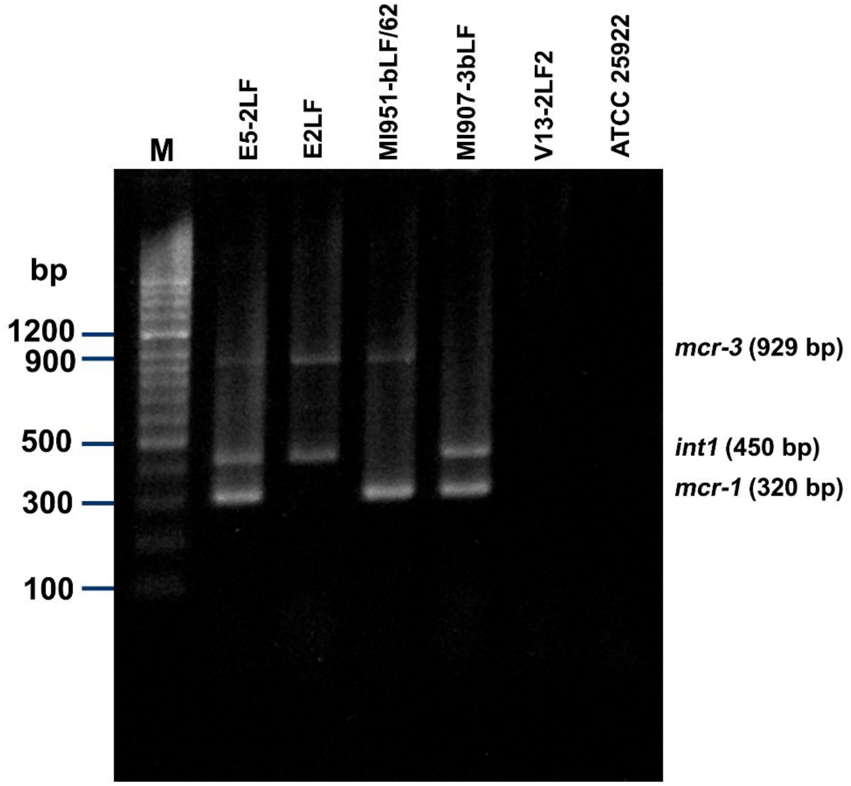

Figure S1. Presence of *mcr-1*/*mcr-3* in *E. coli* strains used in this study. *E. coli* ATCC 25922 and V13-2LF2 were used as the negative controls. *mcr-1* and *-3* and integron-associated (*int1*) genes were detected in *E. coli* MI907-3bLF, MI951-bLF/62, E2LF, and E5-2LF by Multiplex PCR

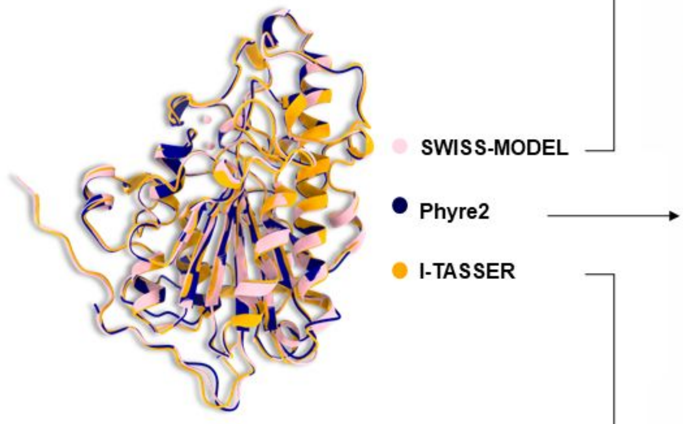

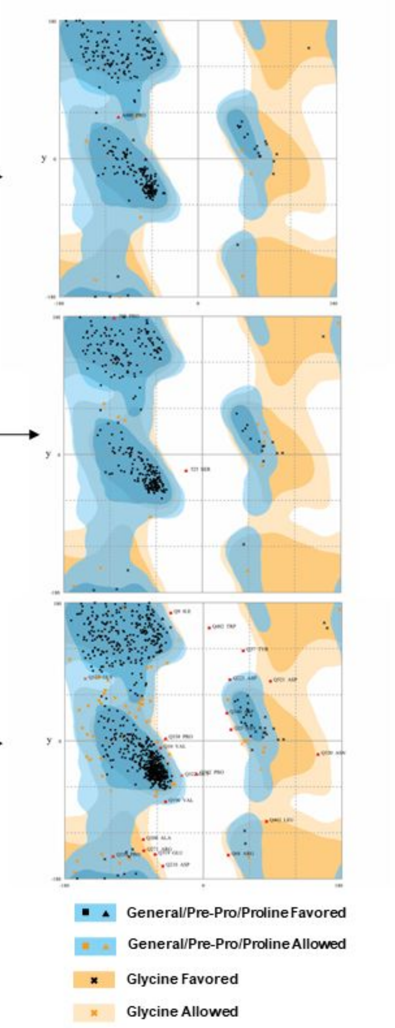

		Ramachandran by RAMPAGE		
	Template similarity	favored region (%)	allowed region (%)	outlier region (%)
SWISS-MODEL	4kay (61.61%)	97.3	2.4	0.3
Phyre2	4kay (61.61%)	95.7	3.7	0.6
I-TASSER	4kay (61.61%)	90.9	6.7	2.4

Figure S2. Homology Models of MCR-3. Homology models shown are results from SWISS-MODEL (pink), Phyre2 (navy), and I-TASSER (orange) using the *Neisseria meningitidis* LptA (a PEA transferase; PDB id 4KAY)[1] as template, with Ramachandran plots analyzed through the RAMPAGE server [2].

(A)

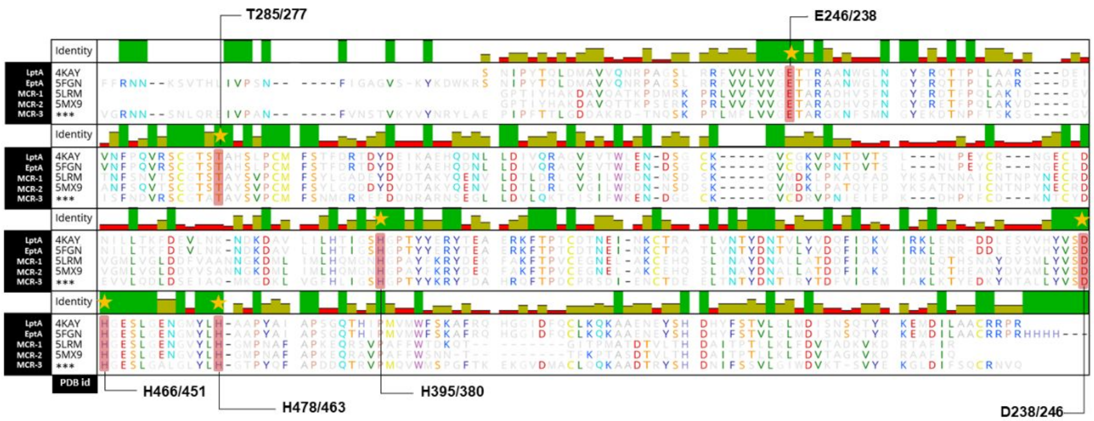

(B)

Identity	MCR-1 (PDB id: 5lrm)	MCR-2 (PDB id: 5mX9)	MCR-3 (ASF81896.1)	EptA (PDB id: 5FGN)	LptA (PDB id: 4KAY)
MCR-1		81.30%	35.30%	36.80%	42.00%
MCR-2	88.90%		35.00%	35.70%	42.00%
MCR-3	53.80%	54.10%		38.50%	44.80%
5FGN	52.70%	53.00%	58.60%		100.00%
4KAY	55.70%	57.60%	63.00%	100.00%	
similarity	MCR-1	MCR-2	MCR-3	5FGN	4KAY

Figure S3 Analysis of selected PEA transferases from Protein Data Bank. (A), Multiple sequence alignment of representative sequences of LptA, EptA, MCR-1, MCR-2 (pdb accessions as shown) and MCR-3 (sequence accession No. WP_039026394). (B) Identity and similarity alignment by EMBOSS Water [3] between MCR-1, MCR-2, MCR-3**,** LptA and EptA.

(A)

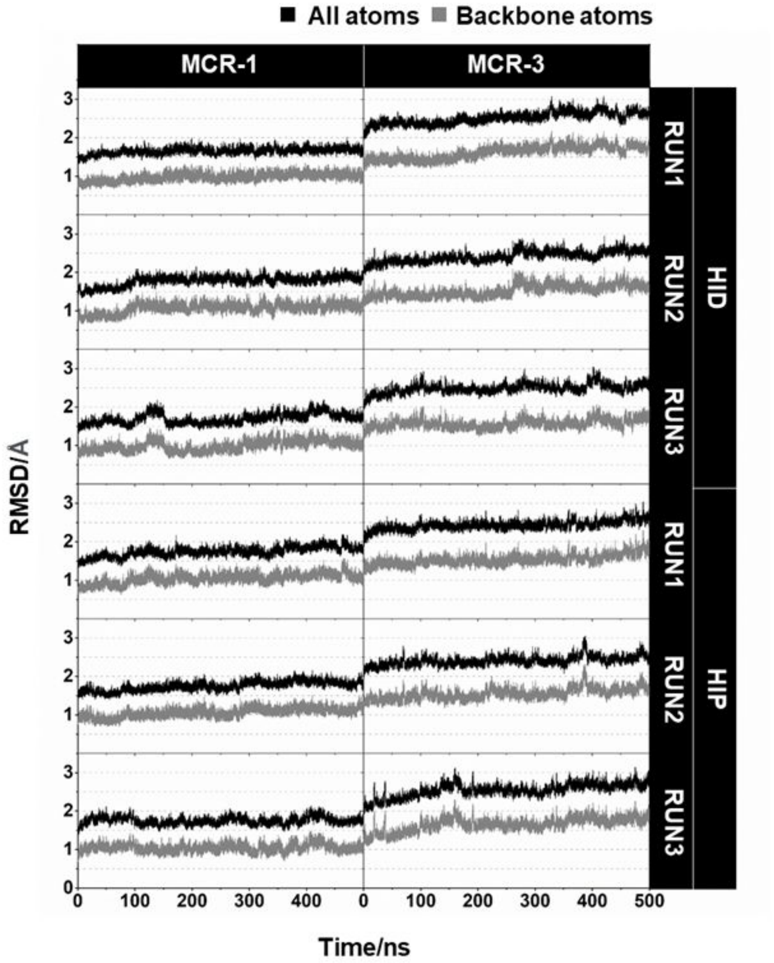

All atoms
Backbone atoms
MCR-1
MCR-3
RUN1
RUN2
RUN3
RUN1
RUN2
RUN3
HID
HIP
RMSD/Å
0
1
2
3
0
100
200
300
400
500
Time/ns

(B)

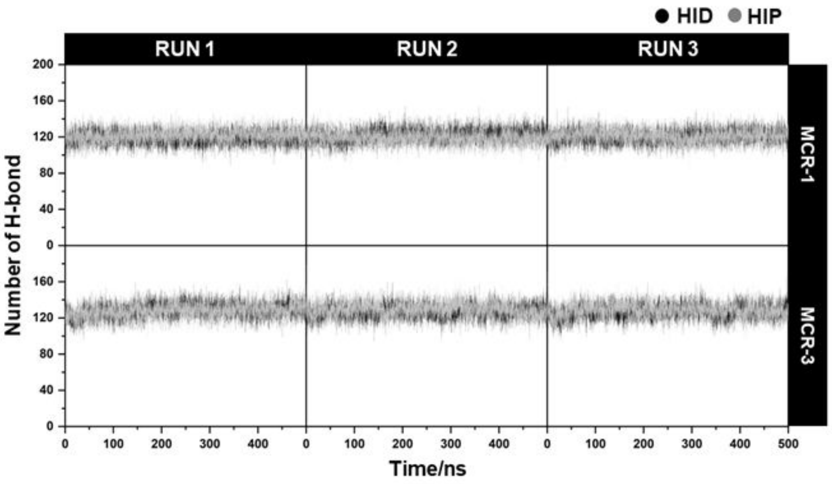

HID
HIP
RUN 1
RUN 2
RUN 3
MCR-1
MCR-3
Number of H-bond
0
40
80
120
160
200
0
100
200
300
400
500
Time/ns

Figure S4. Stabilities of MCR-1/MCR-3 over 500 ns Molecular Dynamic Simulations. (A) RMSD values (compared to starting structures), (B) Numbers of H-bonds for MCR-1/MCR-3 structures each in three independent systems with different initial velocities.

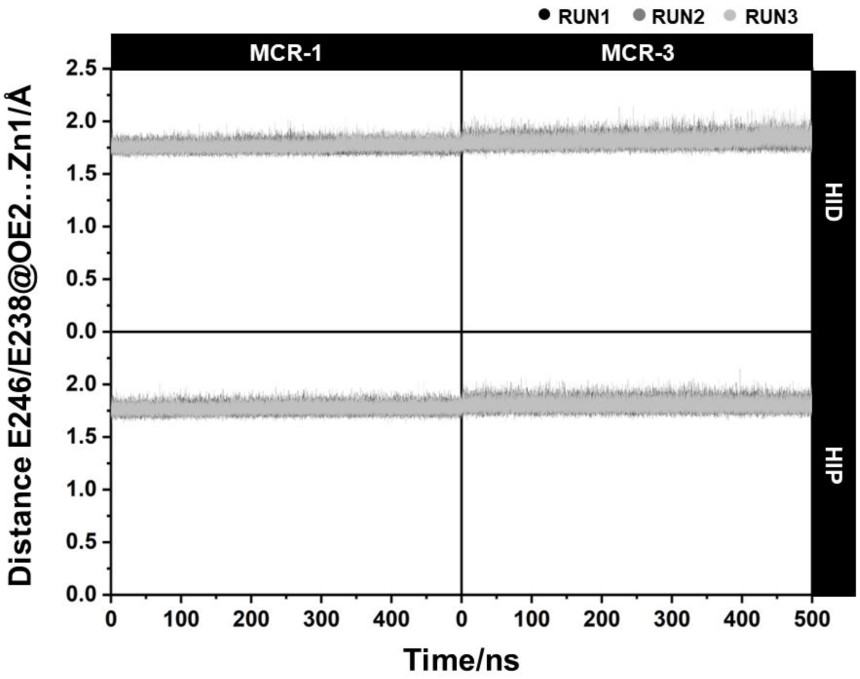

Figure S5. Stability of Zn1 Coordination over 500 ns MD Simulations of MCR-1/MCR-3. Plots show the distance between the OE2 oxygen atoms of E246/E238 in MCR-1/MCR-3, respectively, and Zn1.

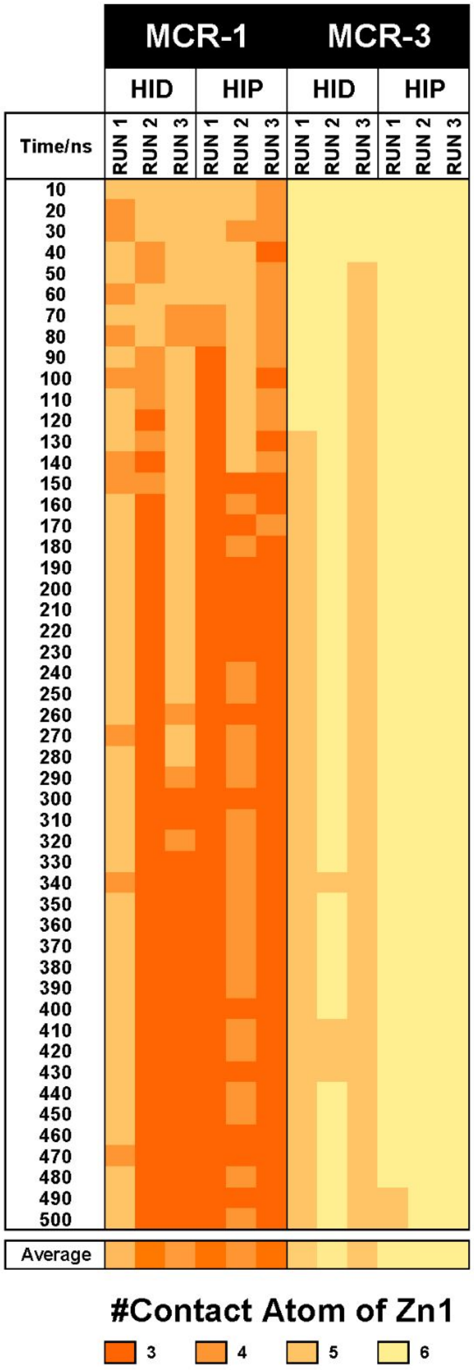

Figure S6. Zn1 Coordination over 500 ns MD Simulations of MCR-1/MCR-3. Time evolution of residues within 2.7 Å of the Zn1 atom, where color boxes represent the number of contacting atoms.

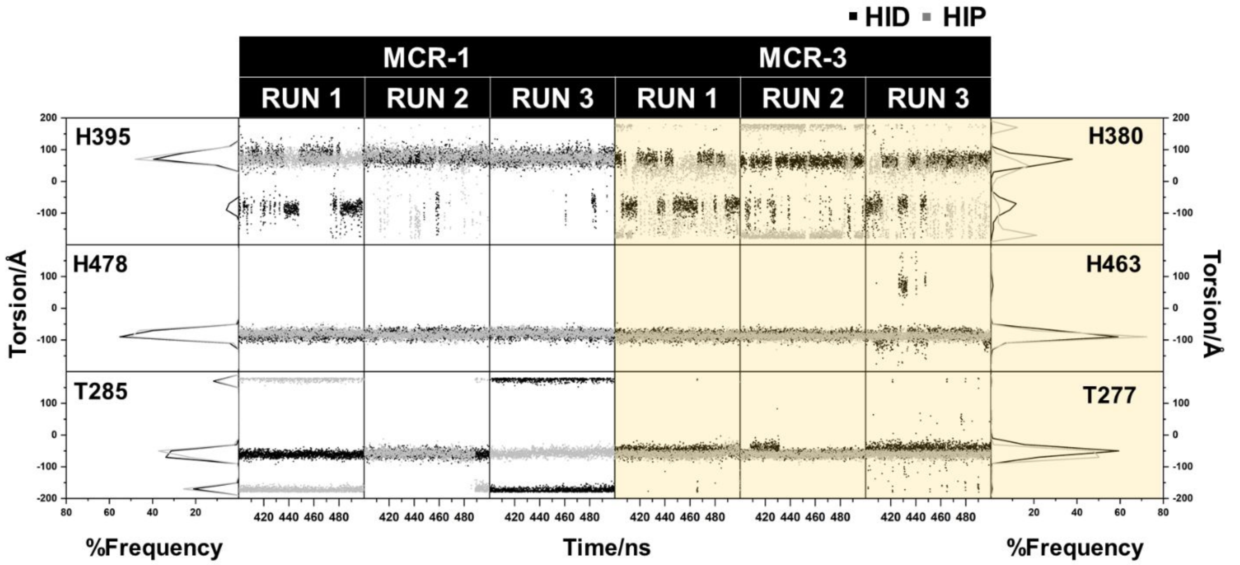

Figure S7. Rotational Flexibility of Selected Active Site Residues over 500 ns MD Simulations of MCR-1/MCR-3. Distributions of torsion angles of H395/H380, H478/H463 and T285/T277 for 500 ns molecular dynamics simulations of MCR-1/MCR-3.

(A)

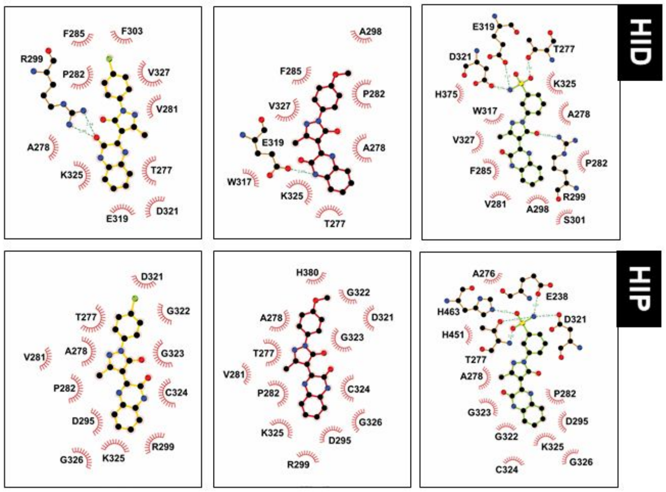

		ST3c (Ref.)		ST3e		ST3f	
		HID	HIP	HID	HIP	HID	HIP
ACTIVE SITE RESIDUE	E238						✓
	T277	✓	✓	✓	✓	✓	✓
	H380				✓		
	D450						
	H451						✓
	H463						✓
	A276					✓	✓
	A278	✓	✓	✓	✓		✓
	V281	✓	✓		✓	✓	
	P282	✓	✓	✓	✓	✓	✓
	F285	✓		✓		✓	
	D295		✓		✓		✓
	A298			✓		✓	
	R299	✓	✓		✓	✓	
	S301					✓	
	F303	✓					
	W317			✓		✓	
	E319	✓		✓		✓	
	D321	✓	✓		✓	✓	✓
	G322		✓		✓		✓
	G323		✓		✓		
	C324		✓		✓		✓
	K325	✓	✓	✓	✓	✓	✓
	G326				✓		✓
	V327	✓		✓		✓	
	H375					✓	

(B)

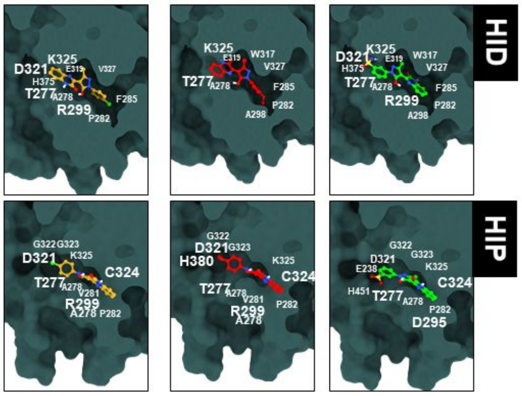

Figure S8. Interactions of Pyrazolones Docked into MCR-3. (A) Ligplot analyses of docked poses of pyrazolones (Ref, ST3e, and ST3f) into the MCR-3 active site showing hydrophobic and hydrogen bond interactions, interacting residues shown in table (right). (B) binding poses overlaid on space-filling model of MCR-3 active site.

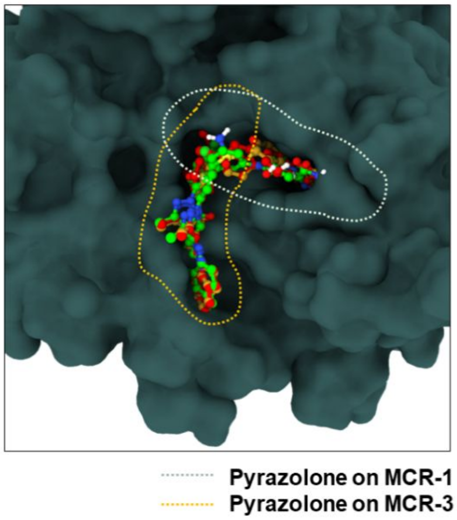

Figure S9 Comparison of Pyrazolone Binding Poses to MCR-1 and MCR-3. Structures (ref, ST3e and ST3f) shown are selected low-energy poses from docking results.

Table S1. The MIC of *Escherichia coli* ATCC 25922, V13-2LF2 (negative control), MI907-3bLF (*mcr-1*), MI951-bLF/62 (*mcr-3*) E2LF and E5-2LF (*mcr-1, mcr-3*) that carry the *mcr-1* and *mcr-3* gene and pyrazolone compounds 0.0625-64 µg/mL (2-fold dilution) in *E. coli.*

	**MIC in presence of compound**		
**Strain**	**Reference compound**	**ST3f**	**ST3e**
E. coli ATCC 25922 (negative control)	> 64	> 64	> 64
E. coli V13-2LF2 (negative control)	> 64	> 64	> 64
E. coli MI907-3bLF (*int1*$^{+}$, *mcr-1*$^{+}$)	> 64	> 64	> 64
E. coli MI951-bLF/62 (*mcr-1*$^{+}$, *mcr-3*$^{+}$)	> 64	> 64	> 64
E. coli E2LF (*int1*$^{+}$, *mcr-3*$^{+}$)	> 64	> 64	> 64
E. coli E5-2LF (*int1*$^{+}$, *mcr-1*$^{+}$, *mcr-3*$^{+}$)	> 64	> 64	> 64

Table S2. Average binding free energies (calculated from 3 replicate docking runs per structure) for Autodock4 docking of pyrazolone compounds to structures (20 for each system) derived from MD simulations of MCR-1/MCR-3 in the HID and HIP states. Colors are as in Figure 5.

	MCR-1		MCR-3	
	HID	HIP	HID	HIP
ETA	-4.4 ± 0.3	-4.6 ± 0.3	-4.6 ± 0.3	-4.5 ± 0.3
Ref.	-6.5 ± 0.6	-6.5 ± 0.7	-6.6 ± 0.7	-6.6 ± 0.6
ST3a	-6.4 ± 0.5	-6.3 ± 0.6	-6.4 ± 0.6	-6.6 ± 0.6
ST3b	-6.8 ± 0.6	-6.7 ± 0.7	-6.7 ± 0.7	-6.8 ± 0.6
ST3d	-6.5 ± 0.6	-6.5 ± 0.6	-6.6 ± 0.6	-6.7 ± 0.6
ST3e	-6.5 ± 0.6	-6.4 ± 0.7	-6.4 ± 0.7	-6.5 ± 0.6
ST3f	-7.1 ± 0.7	-7.2 ± 0.8	-7.1 ± 0.6	-7.1 ± 0.7
ST3g	-5.7 ± 0.4	-6.4 ± 3.1	-5.7 ± 0.6	-5.9 ± 0.5
ST3h	-5.8 ± 0.5	-6.7 ± 4	-5.8 ± 0.5	-5.9 ± 0.4
ST3i	-6.4 ± 0.5	-6.4 ± 0.6	-6.5 ± 0.7	-6.6 ± 0.6
ST3j	-6.4 ± 0.5	-6.4 ± 0.6	-6.4 ± 0.7	-6.5 ± 0.6
ST4a	-5.4 ± 0.5	-6.2 ± 3.1	-5.7 ± 0.6	-5.8 ± 0.5
ST4b	-5.6 ± 0.5	-6.2 ± 2.6	-5.9 ± 0.6	-6.1 ± 0.7
ST4c	-5.6 ± 0.5	-6.4 ± 3	-6 ± 0.6	-6 ± 0.6
ST4d	-6.1 ± 0.5	-6.9 ± 2.9	-6.3 ± 0.6	-6.8 ± 0.8
ST4e	-5.4 ± 0.5	-6.2 ± 2.8	-5.7 ± 0.5	-5.8 ± 0.6
ST4f	-5.6 ± 0.4	-6.5 ± 2.8	-5.8 ± 0.6	-6 ± 0.6
ST4g	-5.8 ± 0.5	-6.5 ± 3.1	-6 ± 0.6	-6.1 ± 0.5
ST4h	-5.9 ± 0.4	-6.4 ± 3	-5.8 ± 0.6	-5.8 ± 0.5
ST4i	-5.2 ± 0.4	-5.3 ± 1.2	-5.2 ± 0.6	-5.4 ± 0.5
ST4j	-5.3 ± 0.5	-5.3 ± 1.2	-5.3 ± 0.6	-5.4 ± 0.6
ST4k	-5.4 ± 0.5	-5.4 ± 1.2	-5.5 ± 0.7	-5.6 ± 0.6

Table S3. The colistin MIC of *Escherichia coli* ATCC 25922, V13-2LF2 (negative control), MI907-3bLF (*mcr-1*), MI951-bLF/62 (*mcr-3*) E2LF and E5-2LF (*mcr-1, mcr-3*) that carry the *mcr-1* and *mcr-3* gene and pyrazolone compounds 8 µg/mL in *E. coli.*

Strain	Colistin MIC (µg/mL)	Colistin MIC in presence of compound (8 µg/mL)		
		ST3b	ST4g	ST3j
E. coli ATCC 25922 (negative control)	0.5*	1	1	1
E. coli V13-2LF2 (negative control)	0.5	1	1	1
E. coli MI907-3bLF (*int1*$^{+}$, *mcr-1*$^{+}$)	8	4	8	4
E. coli MI951-bLF/62 (*mcr-1*$^{+}$, *mcr-3*$^{+}$)	8	4	4	8
E. coli E2LF (*int1*$^{+}$, *mcr-3*$^{+}$)	4	4	4	4
E. coli E5-2LF (*int1*$^{+}$, *mcr-1*$^{+}$, *mcr-3*$^{+}$)	8	8	4	8

References

1. Wanty, C.; Anandan, A.; Piek, S.; Walshe, J.; Ganguly, J.; Carlson, R. W.; Stubbs, K. A.; Kahler, C. M.; Vrielink, A., The Structure of the *Neisserial* Lipooligosaccharide Phosphoethanolamine Transferase A (LptA) Required for Resistance to Polymyxin. *J. Mol. Biol.* **2013,** *425*, 3389-3402.
2. Lovell, S. C.; Davis, I. W.; Arendall, W. B., 3rd; de Bakker, P. I.; Word, J. M.; Prisant, M. G.; Richardson, J. S.; Richardson, D. C., Structure validation by Calpha geometry: phi,psi and Cbeta deviation. *Proteins* **2003,** *50*, 437-50.
3. Smith, T. F.; Waterman, M. S., Identification of common molecular subsequences. *J. Mol. Biol.* **1981,** *147*, 195-197.
